# Acute Infections and Inflammatory Biomarkers in Patients with Acute Pulmonary Embolism

**DOI:** 10.3390/jcm12103546

**Published:** 2023-05-18

**Authors:** Ann-Sophie Eggers, Alaa Hafian, Markus H. Lerchbaumer, Gerd Hasenfuß, Karl Stangl, Burkert Pieske, Mareike Lankeit, Matthias Ebner

**Affiliations:** 1 Department of Cardiology, Angiology and Intensive Care Medicine, Charité Campus Virchow-Klinikum Mittelallee, German Heart Center of the Charité—University Medicine Berlin, 13353 Berlin, Germany; 2 German Center for Cardiovascular Research (DZHK), Partner Site Berlin, 10785 Berlin, Germany; 3 Clinic of Cardiology and Pneumology, University Medical Center Göttingen, 37075 Goettingen, Germany; 4 Department of Radiology, Campus Charité Mitte (CCM), Charité—University Medicine Berlin, 10117 Berlin, Germany; 5 German Center for Cardiovascular Research (DZHK), Partner Site Goettingen, 37075 Goettingen, Germany; 6 Department of Cardiology, Angiology and Intensive Care Medicine, Charité Campus Mitte, German Heart Center of the Charité—University Medicine Berlin, 10117 Berlin, Germany; 7 Independent Researcher, 14195 Berlin, Germany

**Keywords:** infection, pulmonary embolism, CRP, PCT, outcome

## Abstract

Although infections are frequent in patients with pulmonary embolism (PE), its effect on adverse outcome risk remains unclear. We investigated the incidence and prognostic impact of infections requiring antibiotic treatment and of inflammatory biomarkers (C-reactive protein [CRP] and procalcitonin [PCT]) on in-hospital adverse outcomes (all-cause mortality or hemodynamic insufficiency) in 749 consecutive PE patients enrolled in a single-centre registry. Adverse outcomes occurred in 65 patients. Clinically relevant infections were observed in 46.3% of patients and there was an increased adverse outcome risk with an odds ratio (OR) of 3.12 (95% confidence interval [CI] 1.70–5.74), comparable to an increase in one risk class of the European Society of Cardiology (ESC) risk stratification algorithm (OR 3.45 [95% CI 2.24–5.30]). CRP > 124 mg/dL and PCT > 0.25 µg/L predicted patient outcome independent of other risk factors and were associated with respective ORs for an adverse outcome of 4.87 (95% CI 2.55–9.33) and 5.91 (95% CI 2.74–12.76). In conclusion, clinically relevant infections requiring antibiotic treatment were observed in almost half of patients with acute PE and carried a similar prognostic effect to an increase in one risk class of the ESC risk stratification algorithm. Furthermore, elevated levels of CRP and PCT seemed to be independent predictors of adverse outcome.

## 1. Introduction

Pulmonary embolism (PE) is associated with high morbidity and mortality, making it a major contributor to global disease burden [1,2,3]. As the individual patient risk for PE-related adverse outcome varies widely, current clinical practice guidelines stress the importance of early risk stratification to guide therapeutic decision making [2]. The main pillars of risk assessment in acute PE consist of clinical parameters reflecting the haemodynamic status of the patient, laboratory parameters indicating myocardial injury and imaging of right ventricle (RV) to detect signs of RV dysfunction. In addition, the prognostic role of aggravating conditions and comorbidities, such as concomitant deep vein thrombosis (DVT), cancer and chronic heart failure, is well established and requires consideration when evaluating a patient’s overall adverse outcome risk [2]. In contrast, even though acute inflammation and infections are frequently observed in patients with PE, their prognostic effect on patient outcome remains insufficiently studied.

Preclinical studies suggest that inflammatory pathways influence the development of acute RV dysfunction, the main determinant of adverse outcome and death in acute PE [4]. However, two small clinical studies that assessed the association between elevated levels of the inflammatory biomarker C-reactive protein (CRP) and clinical outcome of patients with PE provided conflicting results [5,6]. Procalcitonin (PCT), another well-established biomarker of inflammation that predicts outcome in patients with pulmonary infections, has never been investigated for its prognostic role in PE [7]. Furthermore, pneumonia due to PE-related pulmonary infarction is a common complication of PE, affecting 30 to 70% of patients [8,9,10]. Yet, robust data on the influence of pneumonia and other clinically relevant infections on the likelihood of PE-related adverse outcomes are scarce [7,8,11].

In the present study we investigated the impact of the inflammatory biomarkers CRP and PCT and infections requiring antibiotic treatment on the clinical outcome and on the duration of the in-hospital stay of patients with acute PE.

## 2. Materials and Methods

### 2.1. Study Design and Outcomes

The Pulmonary Embolism Registry of Göttingen (PERGO) prospectively enrolls consecutive patients with objectively confirmed PE ≥ 18 years of age admitted to the University Medical Center Göttingen, Germany. The study protocol has been described in detail previously [12,13]. The present analysis included patients enrolled in PERGO between September 2008 and February 2019. After obtaining informed consent for participation in PERGO, complete data on comorbidities, previous medication, symptoms at the time of PE diagnosis, results of diagnostic tests (including laboratory parameters, ECG, transthoracic echocardiography (TTE) and computed tomography pulmonary angiography (CTPA)), clinical diagnosis of acute infections, antibiotic treatment and clinical outcomes were obtained using a standardised case report form. To identify patients with clinically relevant infections requiring antibiotic treatment we individually reviewed the patient charts of all study patients. In those treated with antibiotics, we further reviewed physician’s letters for the likely clinical cause of infection based on the judgement of the treating physicians. Moreover, TTE and CTPA images were reviewed by an independent investigator to obtain parameters defining RV dysfunction.

We excluded patients (1) with missing CRP measurements at the time of PE diagnosis, (2) with cardiac arrest as the presenting symptom, (3) with other acute severe cardiopulmonary or infectious disease responsible for clinical presentation and symptoms and (4) patients with recurrent PE, who had been previously enrolled in PERGO (only the first event was included in the analysis). All patients were followed for the clinical course of the in-hospital stay.

Diagnostic and therapeutic management was in accordance with the European Society of Cardiology (ESC) 2008 (September 2008–August 2014) and 2014 (September 2014–February 2019) PE guidelines and local standard operating procedures [14,15]. All related decisions were left to the discretion of the treating physicians and not influenced by the study protocol. The study was conducted in accordance with the amended Declaration of Helsinki and approved by the local independent Ethic Committee of the University Medical Center Göttingen, Germany (protocol number 14/6/10). All patients gave informed written consent for participation in the study.

Tachycardia was defined as a heart rate of ≥100 beats per minute (bpm) and hypoxia as a peripheral oxygen saturation < 90%. Fever was defined as a body temperature ≥ 38.0 °C and was measured using a tympanic thermometer at the time of pulmonary embolism diagnosis. Renal insufficiency was defined as a glomerular filtration rate (GFR) < 60 mL/min/1.73 m^2^ body surface area. Altered mental status was defined as disorientation, somnolence, sopor or coma. Active cancer was defined as known malignant disease, treatment with antitumour therapy within the last 6 months, metastatic state or haematologic cancer not in complete remission [16]. RV dysfunction on CTPA was defined as right-to-left ventricular (RV/LV) diameter ratio ≥ 1.0 [3]. Patients were stratified to risk classes post-hoc according to the algorithm proposed by the ESC 2019 guidelines and the quick Sequential Organ Failure Assessment (qSOFA) score [2,17,18]. For calculation of all algorithms and scores, missing values were considered to be normal [19].

The primary study outcome was defined as in-hospital all-cause mortality or hemodynamic insufficiency (cardiopulmonary resuscitation or administration of catecholamines) and is referred to as in-hospital adverse outcome in the manuscript. Other investigated endpoints include in-hospital all-cause mortality, the median duration of in-hospital stay and intubation. All events and causes of death were independently adjudicated by two of the authors (M.E. and A.E.) and disagreement was resolved by a third author (M.L.). 

### 2.2. Biomarker Measurements

Venous blood samples were collected within 6 h of the time of PE diagnosis, processed using standard operating procedures and immediately stored at −80 °C. C-reactive protein (MULTIGENT CRP VARIO, Abbott Laboratories, Wiesbaden, Germany) was measured by the laboratory of the University Medical Center Göttingen, Germany. Plasma concentrations of procalcitonin (PCT, BRAHMS, Roche Diagnostics, Mannheim, Germany) was measured in batches after a single thaw by the amedes MVZ wagnerstibbe laboratory in Göttingen, Germany. 

For CRP, the following predefined cut-off values were investigated: (1) >5 mg/L (upper limit of normal) and (2) >50 mg/L (previously described cut-off for risk stratification in acute PE) [5,20]. Cut-off values indicating elevated levels for PCT were defined as following: (1) PCT > 0.07 µg/L (upper limit of normal) and (2) PCT > 0.25 µg/L (established cut-off for initiation of antibiotic treatment in pneumonia) [21,22].

### 2.3. Statistical Analysis

Categorical variables are presented as total numbers and percentages and continuous variables are presented as medians with the corresponding interquartile range (IQR). Categorical variables were compared using the chi-squared, Fisher’s exact test and the Mantel–Haenszel test of trend, as appropriate. The Mann–Whitney U-test was used to compare continuous variables. 

The area under the curve (AUC) of prognostic relevant laboratory parameters with regard to study outcomes was determined using receiver operating characteristic (ROC) analysis with corresponding 95% confidence intervals (CI). The lowest concentration of each inflammatory biomarker providing >90% specificity was selected as a cohort optimised cut-off value. To further evaluate cohort optimised cut-off values and predefined cut-off values, specificity, sensitivity, negative and positive predictive values as well as the negative and positive likelihood ratios were calculated. 

Additionally, the prognostic value of comorbidities, patient characteristics and biomarkers was tested using univariable and multivariable logistic regression analysis and results are presented as odds ratios (ORs) with the corresponding 95% CI. To confirm the independent prognostic value of CRP and PCT, levels were dichotomized at the optimal cut-off value and entered into multivariable logistic regression models correcting for (I) presence of hypotension on admission, (II) elevated troponin levels (hsTnT ≥ 14 pg/mL), (III) risk class according to the ESC 2019 risk stratification algorithm and (IV) simplified Pulmonary Embolism Severity Index (sPESI) class [2,23]. The independent prognostic value of clinically relevant infections was tested in a multivariable model that included prognostically relevant comorbidities (based on univariable analyses) and ESC risk class. 

All tests were two-sided and significant findings was defined by a *p*-value < 0.05. As this was explorative testing, no adjustments for multiple testing were carried out. P-values were provided for descriptive reasons only and should be interpreted with caution and in connection with effect estimates. Statistical analysis was performed through Statistics Package for Social Sciences (IBM SPSS Statistics, Version 27, IBM Corp. Armonk, NY, USA).

## 3. Results

Between September 2008 and February 2019, 1027 patients were enrolled in PERGO. Exclusion criteria applied to (1) 125 (12.2%) with missing C-reactive protein (CRP) measurement at the time of PE diagnosis, (2) 43 (4.2%) patients with cardiac arrest as the presenting symptom, (3) 96 (9.3%) with other acute severe cardiopulmonary or infectious disease and (4) 14 (1.4%) patients with PE recurrence, who were already enrolled in PERGO. Hence, 749 (72.9%) patients were included in the present analysis. 

At presentation, 81 (10.8%) patients were classified as low risk, 366 (48.9%) as intermediate-low risk, 266 (35.5%) as intermediate-high risk and 36 (4.8%) as high risk according to the 2019 ESC risk stratification algorithm. The median level of CRP at presentation was 33.5 (IQR 11.4-71.2) mg/L and elevations above the upper limit of normal (>5 mg/L) were observed in 86.4% of patients. Data on procalcitonin levels were available for 538 patients (71.8%). Of these, only 34.2% had concentrations above the upper limit of normal (>0.07 µg/L; median concentration 0.05 [IQR 0.03–0.10] µg/L). Patients with PCT elevations above the upper level of normal had higher rates of tachycardia (47.7% vs. 27.1%; *p* < 0.001), hypotension (9.4% vs. 4.3%; *p* = 0.023), hypoxia (29.9% vs. 19.4%; *p* = 0.013) and leukocytosis (45.8% vs. 33.7%; *p* = 0.007) compared to patients without elevated PCT. Moreover, patients with PCT elevations at admission were more frequently diagnosed with pneumonia (42.9% vs. 30.8%; *p* = 0.005) during the further inpatient course. Higher PCT levels were also reported in patients with renal insufficiency (*n* = 370) compared to patients without renal insufficiency (0.08 μg/L [IQR 0.05–0.17] vs. 0.04 μg/L [IQR 0.03–0.08]; *p* < 0.001).

An in-hospital adverse outcome occurred in 65 (8.7%) patients. Of the 26 patients who died during the in-hospital stay, there were 17 (65.4%) deaths due to PE, 6 (23.1%) due to infections and 3 (11.5%) due to cancer. Further information on comorbidities, initial presentation and outcomes is shown in Table 1, left column. 

Patients receiving antibiotic treatment during the first 7 days after PE more frequently presented with fever, had higher levels of CRP and PCT and a higher leucocyte count compared to patients without antibiotic treatment (*p* < 0.001 for each comparison; Table 1, right columns).

### 3.1. Frequency of Infections Requiring Antibiotic Treatment

Overall, 347 (46.3%) patients required antibiotic treatment during the first seven days after PE. In 302 (87.0%) patients, antibiotic treatment was initiated after PE diagnosis (median time to treatment start 0 [IQR 0–1] days); 45 (13.0%) patients were already under antibiotic treatment at the time of PE diagnosis. 

**Figure 1:**
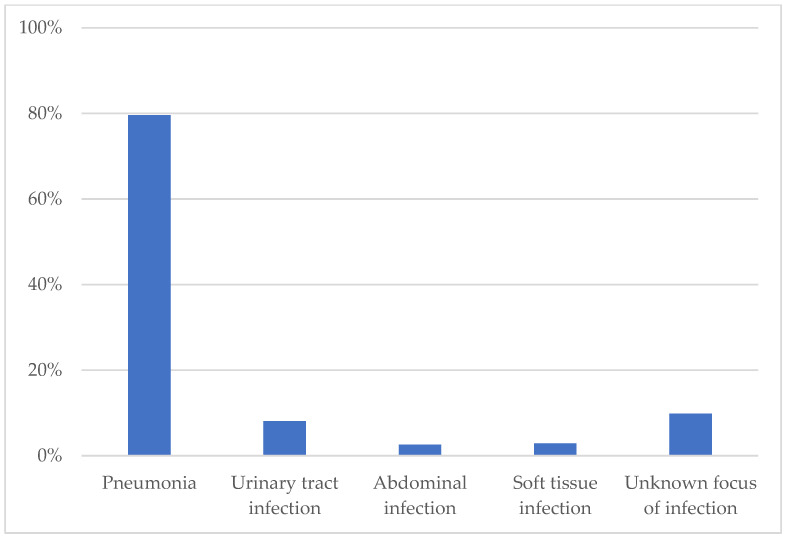
Indications for antibiotic treatment.

As shown in Figure 1, the most frequent clinical cause for antibiotic treatment was pneumonia (79.6%), followed by infections of the urinary tract (8.1%), soft tissue (2.9%), abdomen (2.6%) and infections of unknown origin (9.8%). Data on the employed antibiotic classes are provided in Table S1 of the Online Supplement.

Importantly, results of the sPESI score and the ESC 2019 risk stratification algorithm did not differ between patients with and without antibiotic treatment during the first 7 days after PE diagnosis (*p* = 0.87 and *p* = 0.11, respectively; Table 1, right columns).

### 3.2. Prognostic Impact of Clinical Infections Requiring Antibiotic Treatment

Patients requiring antibiotic treatment due to acute infections had an OR of 3.08 (95% CI 1.77–5.37) for developing an in-hospital adverse outcome (Table 2). A similar prognostic effect was observed in in the subgroup of patients with antibiotic treatment for pneumonia (OR 3.52 [95% CI 1.95–6.37]), when compared to patients with no antibiotic treatment. Furthermore, no relevant differences were observed between patients with established antibiotic treatment at the time of PE diagnosis (OR 4.36 [95% CI 1.79–10.64]) and patients with new antibiotic treatment initiated within 7 days after PE (OR 2.90 [95% CI 1.64–5.14]). Patients who required antibiotic treatment were also more likely to be intubated (OR 4.44 [95% CI 2.01–9.79]), a finding that was not more pronounced in the subgroup of patients with pneumonia (OR 4.65 [95% CI 2.06–10.46]).

In a multivariable logistic regression analysis that included antibiotic treatment, other prognostically relevant comorbidities and ESC risk class, antibiotic treatment was found to be an independent predictor of in-hospital adverse outcome (OR 3.12 [95% CI 1.70–5.74]; Table 2). The observed prognostic effect was comparable in magnitude to an increase in one risk class of the ESC 2019 algorithm (OR 3.45 [95%CI 2.24–5.30]). Furthermore, patients requiring antibiotic treatment had a longer duration of in-hospital stay compared to patients with no antibiotic treatment (11.0 [IQR 6.0–16.0] vs. 7.0 [IQR 4.0–11.0] days, *p* < 0.001).

### 3.3. Prognostic Impact of Biomarkers of Inflammation

**Figure 2:**
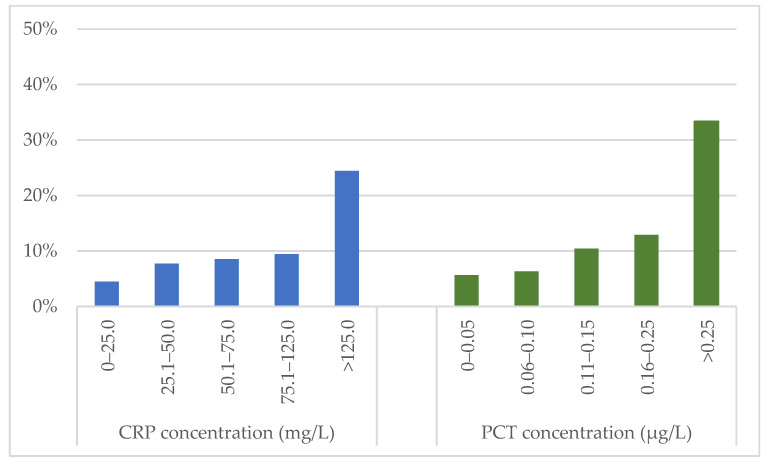
Frequency of in-hospital adverse outcome in patients stratified according to CRP and PCT concentrations at admission. CRP: C-reactive protein; PCT: procalcitonin.

Median concentrations of CRP and PCT at the time of PE diagnosis were higher in patients who developed an in-hospital adverse outcome compared to patients with a favourable clinical course (61.2 [IQR 27.3–164.8] vs. 31.1 [IQR 10.7–66.3] mg/L and 0.11 [IQR 0.05–0.36] vs. 0.05 [IQR 0.03–0.09] µg/L, respectively; *p* < 0.001 for both comparisons). The rate of in-hospital adverse outcomes in patients stratified by CRP and PCT levels at admission is shown in Figure 2.

Using ROC analyses, we calculated an AUC for the prediction of an in-hospital adverse outcome of 0.67 [95% CI 0.60–0.74] for CRP and 0.69 [95% CI 0.61–0.78] for PCT. Specificity > 90% was provided at a cut-off value of 124 mg/L for CRP and 0.18 µg/L for PCT. A comparison of test characteristics for cohort-derived and prespecified cut-off values is provided in Table 3. Owing to the limited specificity of the previously reported CRP cut-off value (50 mg/L) of only 66.2% [95% CI 62.6–69.7], we chose the cut-off value providing >90% specificity in our cohort for all further analyses. For PCT, the cohort-derived cut-off value was similar to the established cut-off value for antibiotic treatment, and hence the latter was selected for all further analyses in the manuscript. 

Using univariable logistic regression analyses, CRP > 124 mg/L and PCT > 0.25 µg/L predicted an in-hospital adverse outcome (OR 4.64 [95% CI 2.52–8.21] and 6.88 [95% CI 3.48–13.59], respectively; Table 3A) as well as all-cause mortality (OR 5.02 [95% CI 2.21–11.45] and 4.86 [95% CI 1.76–13.41], respectively; Table 3B) and the need for intubation (OR 5.29 [95% CI 2.66–10.53] and 8.02 [95% CI 3.60–17.84], respectively). 

In multivariable logistic regression analyses, CRP or PCT elevations above the selected cut-off values predicted an in-hospital adverse outcome independent of sPESI class, elevated troponin and hypotension on admission, with respective ORs of 4.87 (95% CI 2.55–9.33) and 5.91 (95% CI 2.74–12.76) (Table 4). The predictive value of both, CRP and PCT, was independent of ESC 2019 risk class (OR 5.42 [95% CI 2.31–12.72] and 3.90 [95% CI 1.36-11.17], respectively; Table 4). Furthermore, PCT elevations remained predictive for in-hospital adverse outcomes after correcting for renal insufficiency (OR 5.85 [95% CI 2.62–13.04]).

## 4. Discussion

In the present study we evaluated the prognostic impact of inflammatory biomarkers and infections requiring antibiotic treatment on the clinical outcome and on the duration of the in hospital-stay of patients with acute PE. Our findings obtained in a real-world single-centre cohort of 749 PE patients can be summarized as follows: (1) clinically relevant infections were frequent (2) the effect of infections requiring antibiotic treatment on the risk of an in-hospital adverse outcome was comparable to an increase in one risk class of the ESC 2019 risk stratification algorithm (3) elevated levels of CRP at the time of PE diagnosis were observed in the majority of patients, but only high levels (CRP > 124 mg/L) had more than 90% specificity for the prediction of in-hospital adverse outcomes; (4) elevated concentrations of PCT at the time of PE diagnosis were uncommon and even a moderate elevation of PCT > 0.25 µg/L predicted the main study outcome with high accuracy; (5) the prognostic impact of both inflammatory biomarkers was independent of established risk factors for short-term adverse outcome after PE. 

Even though pulmonary infarction is considered one of the most common complications of PE, data on incidence and prognostic impact is surprisingly scarce [11,24]. Pulmonary infarction is caused by an occlusion of a pulmonary artery which leads to an increase of the bronchial arterial inflow resulting in a higher capillary blood flow and extravasation of blood into the alveoli [24]. Subsequently, tissue necrosis and infarction can develop if the alveolar haemorrhage is not resolved [24]. The first report of pulmonary infarction was published by Hampton in 1940 describing an incidence of as high as 70% in patients with PE [8]. More recent reports show an overall prevalence of pulmonary infarction in PE patients of 30%, with higher rates occurring in younger patients [9]. The development of superimposed pneumonia due to pulmonary infarction is a common complication of PE [11]. Different from prior reports, we focused on clinically relevant pulmonary infections requiring antibiotic treatment. Of note, no prior study has evaluated the frequency of antibiotic treatment in patients admitted due to acute PE. Clinically relevant pulmonary infections were observed in more than a third of patients (35.8%) in our cohort, and nearly half of patients (46.3%) received antibiotic treatment for any type of infection during the first 7 days after PE. Our analyses show that infections requiring antibiotic treatment during the first 7 days after PE had a similar prognostic effect as an increase in one risk class of the ESC 2019 algorithm and prolonged the median duration of the in-hospital stay by more than 50% (11 vs. 7 days). Importantly, no difference in prognosis was observed in patients treated with antibiotics due to pneumonia compared to those treated for other causes. 

It is well established that systemic inflammation favours the development of arterial and venous thrombosis [25]. In preclinical studies a close link between coagulation and inflammatory pathways has been demonstrated [25,26]. In the clinical setting, acute infections as well as chronic inflammatory diseases are considered predisposing factors for the development of venous thromboembolism [2,25]. In contrast, the prognostic impact of inflammation on the outcome of patients with an acute PE is insufficiently studied. Experimental models show that inflammatory mediators and cells contribute to the remodelling of RV, resulting in RV dysfunction and failure, the main prognosticators of adverse outcome and death in acute PE [4]. In addition, systemic inflammation leads to vasodilation, resulting in a reduction of peripheral vascular resistance, which may aggravate the haemodynamic insufficiency caused by PE [27]. Clinical data to back up these hypotheses has been largely lacking so far. Two small clinical studies have investigated the prognostic value of CRP in acute PE patients with conflicting results [5,6]. A small study including 56 patients showed higher rates of RV dysfunction and mortality in PE patients with elevated CRP levels [5]. In contrast, a single centre report based on data of 152 normotensive PE patients found no association between CRP levels and mortality [6]. PCT, a well-established biomarker in pulmonary infections, has to our knowledge never been evaluated for its prognostic performance in acute PE patients [28]. The only studies analysing PCT in the context of acute PE showed the potential ability of PCT to differentiate PE from pneumonia in patients presenting with acute dyspnoea [29,30]. 

Our investigation is the first to evaluate the prognostic value of both biomarkers in a large cohort of real-life PE patients. Since we aimed to investigate overall patient outcome rather than just PE-related complications, we chose a composite of all-cause mortality and hemodynamic instability as our primary study outcome. Our study demonstrates that median levels of CRP and PCT were higher in patients who developed hemodynamic insufficiency or death compared to patients with a favourable in-hospital course. High levels of CRP (CRP > 124 mg/L) were found to be associated with an in-hospital adverse outcome, but in contrast to the findings of Abul et al., we observed no association between RV dysfunction and elevated CRP levels [5]. In accordance with previous reports, only a minority of PE patients had concentrations of PCT above the upper limit of normal [29,30]. However, if present, even moderate elevations of PCT (>0.25 µg/L) predicted the occurrence of haemodynamic instability or death with >90% specificity. A multi-centre cohort study by Kruger et al. reported that a similar PCT cut-off value of 0.23 µg/L predicted death in 1671 patients with community-acquired pneumonia [31]. However, nearly one third of our cohort had a renal insufficiency in which former studies showed significantly higher PCT concentrations in patients with chronic kidney disease but without a history of dialysis or infection compared to healthy patients [32,33]. In accordance, our analysis showed higher PCT levels in patients with renal insufficiency compared to patients without renal insufficiency. This did not influence the prognostic value of PCT elevations > 0.25 µg/L, that remained predictive of an adverse outcome even after correcting for renal function.

Importantly, even though our results indicate that patients with CRP and PCT elevations at the time of PE diagnosis have a worse short-term prognosis, these patients were not at higher baseline risk for PE related complications according to the ESC risk assessment algorithm when compared to the rest of the study cohort. In accordance, the predictive value of both, CRP and PCT, was independent of established risk factors of short-term PE-related mortality such as sPESI class, elevated troponin levels and hypotension on admission. This finding might be explained by the increased demand for cardiac output in patients with an acute inflammation. This may aggravate PE-induced RV dysfunction making haemodynamic decompensation more likely. Based on the study findings, we hypothesize that patients with an acute PE and elevated inflammatory biomarkers at the time of PE diagnosis may benefit from close monitoring to allow early detection and treatment of impending haemodynamic deterioration. Hence, we would suggest to routinely measure CRP and PCT in all patients diagnosed with acute PE and closely assess these patients for clinical signs of acute infections during the first days after diagnosis.

### Limitations

The present study is a post hoc analysis of a prospective single-centre cohort limiting the generalisability of study findings. Further, PCT measurements were only available for 71.8% of patients and measurements of PCT and CRP were only performed at the time of PE diagnosis. Future studies should evaluate whether the prognostic value of inflammatory biomarkers can be improved by serial measurements. When interpreting our results, it should be kept in mind that CRP is an acute-phase protein and elevated concentrations do not only occur due to infections but can be triggered by inflammatory reactions of many causes. Furthermore, due to the limitations of a registry analysis, we cannot exclude that antibiotic treatment was initiated inappropriately in some patients. Pulmonary infarction caused by acute PE can mimic pneumonia in both, clinical symptoms and radiological findings, making differentiation in everyday clinical practice difficult. In addition, information to differentiate between community-acquired pneumonia and hospital-acquired pneumonia was not available. Based on the available data, differentiation between septic and cardiogenic shock as the reason of hemodynamic insufficiency was not possible. Finally, the limited number of patients and events may have impaired the ability to detect small but significant differences between study groups.

## 5. Conclusions

The results of the present study suggest that inflammatory biomarkers and acute infections requiring antibiotic treatment might have a prognostic value in patients with acute PE. Elevated levels of CRP and PCT seemed to be associated with an increased risk of in-hospital adverse outcome in PE patients and seemed to be independent outcome predictors even after correcting for established risk markers such as sPESI class, troponin elevation and hypotension. In patients requiring antibiotic treatment due to acute infections, an increased adverse outcome risk comparable to an increase in one risk class of the ESC 2019 risk stratification algorithm was observed. 

## Figures and Tables

**Table 1:** Patient characteristics, results of risk stratification and outcomes of study patients in the study cohort, stratified according to antibiotic treatment within 7 days after PE diagnosis.

	All Patients	Antibiotic Treatment within 7 Days After PE		
		Yes	No	P
** Subjects *n***	749	347	402	
Age ≥ 75 years	255 (34.0%)	118 (34.0%)	137 (34.1%)	0.98
Sex (female)	387 (51.7%)	177 (51.0%)	210 (52.2%)	0.74
** Comorbidities **				
Chronic heart failure	109 (14.6%)	62 (17.9%)	47 (11.7%)	** 0.017 **
Coronary artery disease	128 (17.1%)	59 (17.0%)	69 (17.2%)	0.95
Arterial hypertension	479 (64.0%)	216 (62.2%)	263 (65.4%)	0.37
Chronic pulmonary disease	121 (16.2%)	52 (15.0%)	69 (17.2%)	0.42
Renal insufficiency	238 (31.9%)	112 (32.5%)	126 (31.5%)	0.79
Diabetes mellitus	116 (15.5%)	55 (15.9%)	61 (15.2%)	0.80
Anaemia	251 (33.5%)	132 (38.0%)	119 (29.6%)	** 0.015 **
Active cancer	145 (19.4%)	72 (20.7%)	73 (18.2%)	0.37
** Symptoms at presentation **				
Dyspnea	601 (80.5%); *n* = 747	289 (83.5%); *n* = 346	312 (77.8%); *n* = 401	** 0.049 **
Syncope	103 (13.8%); *n* = 748	41 (11.8%); *n* = 346	62 (15.4%); *n* = 402	0.16
** Clinical findings at presentation **				
Tachycardia (heart rate ≥ 100 /min)	259 (35.1%); *n* = 737	130 (37.6%); *n* = 346	129 (33.0%); *n* = 391	0.19
Hypoxemia (SpO2 <90%)	150 (23.7%); *n* = 634	83 (27.5%); *n* = 302	67 (20.2%); *n* = 332	** 0.031 **
Fever (temperature ≥ 38.0 °C)	32 (5.3%); *n* = 602	25 (8.4%); *n* = 298	7 (2.3%); *n* = 304	** <0.001 **
** Laboratory markers of inflammation **				
CRP (mg/L)	33.5; IQR 11.4-71.2	55.4; IQR 27.7-111.6	17.4; IQR 5.5-42.6	** <0.001 **
CRP > 5 mg/L *	647 (86.4%)	334 (96.3%)	313 (77.9%)	** <0.001 **
PCT (µg/L)	0.05; IQR 0.03-0.10; *n* = 538	0.06; IQR 0.03-0.13; *n* = 245	0.05; IQR 0.03-0.08; *n* = 293	** <0.001 **
PCT > 0.07 µg/L *	184 (34.2%); *n* = 538	108 (44.1%); *n* = 245	76 (25.9%); *n* = 293	** <0.001 **
Leukocyte /μL	9.9; IQR 7.7-12.2; *n* = 742	10.9; IQR 8.6-13.6; *n* = 345	9.2; IQR 7.0-11.0; *n* = 397	** <0.001 **
Leukocyte > 10.500/μL *	300 (40.4%); *n* = 742	184 (53.3%); *n* = 345	116 (29.2%); *n* = 397	** <0.001 **
Lactate (venous) mmol/L	1.6; IQR 1.1-2.5; *n* = 510	1.6; IQR 1.1-2.6; *n* = 243	1.6; IQR 1.2-2.4; *n* = 267	** 0.442 **
** Risk stratification **				
sPESI ≥ 1 points (high-risk class)	505 (67.4%)	235 (67.7%)	270 (67.0%)	0.87
ESC 2019 algorithm				0.11
Low risk	81 (10.8%)	46 (13.3%)	35 (8.7%)	
Intermediate-low risk	366 (48.9%)	156 (45.0%)	210 (52.2%)	
Intermediate-high risk	266 (35.5%)	128 (36.9%)	138 (34.3%)	
High risk	36 (4.8%)	17 (4.9%)	19 (4.7%)	
qSOFA (Score ≥ 2 points)	34 (5.4%)	19 (6.5%)	15 (4.4%)	0.25
**Outcomes**				
In-hospital adverse outcome	65 (8.7%)	46 (13.3%)	19 (4.7%)	** <0.001 **
Catecholamine administration	54 (7.2%)	39 (11.2%)	15 (3.7%)	** <0.001 **
In-hospital all-cause mortality	26 (3.5%)	19 (5.5%)	7 (1.7%)	** 0.005 **
Resuscitation	16 (2.1%)	11 (3.2%)	5 (1.2%)	0.07
In-hospital PE-related mortality	17 (2.3%)	11 (3.2%)	6 (1.5%)	0.12
Intubation	39 (5.2%)	30 (8.6%)	9 (2.2%)	** <0.001 **
Duration of in-hospital stay	8.0; IQR 5.0-13.0	11.0; IQR 6.0-16.0	7.0; IQR 4.0-11.0	** <0.001 **

* upper limit of normal of the respective assay. CRP: C-reactive protein; PCT: procalcitonin; sPESI: simplified Pulmonary Embolism Severity Index; ESC: European Society of Cardiology; qSOFA: quick Sequential Organ Failure Assessment; PE: pulmonary embolism. Bold values denote statistical significance at the *p* < 0.05 level.

**Table 2:** Prognostic impact of patient characteristics, comorbidities and usage of antibiotic treatment.

	In–Hospital Adverse Outcome (*n* = 65/749)	
	Univariable OR (95% CI)	Multivariable OR (95% CI)
Antibiotic treatment	** 3.08 (1.77–5.37) **	**3.12 (1.70–5.74)**
Age ≥ 75 years	1.15 (0.68–1.95)	–
Sex (female)	1.03 (0.62–1.71)	–
Chronic heart failure	** 2.28 (1.26–4.13) **	1.05 (0.53–2.07)
Coronary artery disease	1.24 (0.65–2.35)	–
Arterial hypertension	** 3.37 (1.69–6.73) **	**2.28 (1.07–4.87)**
Chronic pulmonary disease	1.19 (0.62–2.31)	–
Renal insufficiency (GFR < 60 mL/min/1.73 m^2^)	** 3.78 (2.23–6.41) **	**1.98 (1.09–3.61)**
Diabetes mellitus	1.41 (0.74–2.68)	–
Anaemia	** 3.11 (1.85–5.23) **	**2.10 (1.19–3.70)**
Active cancer	1.54 (0.86–2.76)	–
Hypotension on admission	** 10.84 (5.28–22.24) **	–
ESC risk assessment algorithm (per class)	**4.09 (2.75–6.10)**	**3.45 (2.24–5.30)**

OR: odds ratio; CI: confidence interval; GFR: glomerular filtration rate; ESC: European Society of Cardiology. Bold values denote statistical significance at the *p* < 0.05 level.

**Table 3:** Prognostic performance of different cut-off values of inflammatory biomarkers with regard to (**A**) in-hospital adverse outcome and (**B**) in-hospital all-cause mortality.

**A: In–Hospital Adverse Outcome**								
	**Prevalence**	**Adverse Outcome Rate**	**Sensitivity** **(95% CI)**	**Specificity** **(95% CI)**	**PPV** **(95% CI)**	**NPV** **(95% CI)**	**LR+**	**OR** **(95% CI)**
CRP > 50 mg/L (previously reported CRP cut–off value) [5,20]	35.9%	14.1%	58.5% (46.3–69.6)	66.2% (62.6–69.7)	14.1% (10.5–18.8)	94.4% (91.9–96.1)	1.7	2.76 (1.64–4.63)
CRP > 124 mg/L (>90% specificity)	12.0%	24.4%	33.8% (22.9–46.7)	90.1% (87.5–92.1)	24.4% (16.3–34.8)	93.5% (91.2–95.2)	3.4	4.64 (2.52–8.21)
PCT > 0.18 µg/L (>90% specificity)	12.6%	29.4%	40.0% (27.6–53.8)	90.2% (87.2–92.5)	29.4% (19.9–41.1)	93.6% (91–95.5)	4.1	6.11 (3.22–11.58)
PCT > 0.25 µg/L (cut–off for antibiotics in pneumonia) [21,22]	9.5%	33.3%	34% (22.4–47.8)	93.0% (90.4–95)	33.3% (22–47)	93.2% (90.6–95.1)	4.9	6.88 (3.48–13.59)
**B: In–hospital all–cause mortality**								
	**Prevalence**	**All–Cause** **Mortality Rate**	**Sensitivity** **(95% CI)**	**Specificity** **(95% CI)**	**PPV** **(95% CI)**	**NPV** **(95% CI)**	**LR+**	**OR** **(95% CI)**
CRP >50 mg/L (previously reported CRP cut–off value) [5,20]	35.9%	5.9%	61.5% (42.5–77.6)	65% (61.5–68.4)	5.9% (3.7–9.4)	97.9% (96.2–98.9)	1.8	2.97 (1.33–6.45)
CRP > 124 mg/L (>90% specificity)	12.0%	11.1%	38.5% (20.1–59.3)	88.9% (86.4–91.1)	11.1% (5.7–19.9)	97.6% (96.0–98.6)	3.5	5.02 (2.21–11.45)
PCT > 0.18 µg/L (>90% specificity)	12.6%	10.3%	36.8% (19.1–59)	88.2% (85.2–90.7)	10.3% (5.1–19.8)	97.4% (95.6–98.5)	3.1	4.38 (1.66–11.55)
PCT > 0.25 µg/L (cut–off for antibiotics in pneumonia) [21,22]	9.5%	11.8%	31.6% (15.4–54)	91.3% (88.6–93.5)	11.8% (5.5–23.4)	97.3% (95.5–98.4)	3.6	4.86 (1.76–13.41)

CI: confidence interval; PPV: positive predictive value; NPV: negative predictive value; LR+: positive likelihood ratio; OR: odds ratio; CRP: C-reactive protein; PCT: procalcitonin.

**Table 4:** Prognostic impact of inflammatory biomarkers and established predictors of short-term mortality.

	In–Hospital Adverse Outcome (*n* = 65/749)			In–Hospital All–Cause Mortality (*n* = 26/749)		
	Univariable OR (95% CI)	Multivariable Model 1 OR (95% CI)	Multivariable Model 2 OR (95% CI)	Univariable OR (95% CI)	Multivariable Model 1 OR (95% CI)	Multivariable Model 2 OR (95% CI)
Laboratory markers of inflammation						
CRP > 124 mg/L	**4.64 (2.52–8.21)**	**4.87 (2.55–9.33)**	–	**5.02 (2.21–11.45)**	**5.42 (2.31–12.72)**	–
PCT > 0.25 µg/L	**6.88 (3.48–13.59)**	–	**5.91 (2.74–12.76)**	**4.86 (1.76–13.41)**	–	**3.90 (1.36–11.17)**
Established risk factors of short–term adverse outcome after PE						
sPESI class	**5.25 (2.23–12.33)**	**3.13 (1.19–8.25)**	**3.04 (1.01–9.15)**	**12.66 (1.7–93.96)**	–	–
hsTnT ≥ 14 pg/mL	**5.7 (2.42–13.43)**	**3.72 (1.52–9.09)**	2.34 (0.93–5.91)	**6.72 (1.57–28.76)**	–	–
RV/LV > 1	2.32 (0.75–7.14)	–	–	0.76 (0.12–4.65)	–	–
Hypotension on admission	**10.84 (5.28–22.24)**	**8.61 (3.79–19.56)**	**6.80 (2.83–16.35)**	**3.93 (1.28–12.07)**	–	–
ESC 2019 risk stratification algorithm (per class)	** 4.09 (2.75–6.10) **	–	–	**3.12 (1.78–5.45)**	**3.36 (1.86–6.08)**	**2.93 (1.54–5.57)**

OR: odds ratio; CI: confidence interval; CRP: C-reactive protein; PCT: procalcitonin; sPESI: simplified Pulmonary Embolism Severity Index; hsTnT: high-sensitivity troponin T; RV/LV: right ventricular-to-left ventricular ratio; ESC: European Society of Cardiology. Bold values denote statistical significance at the *p* < 0.05 level.

## Data Availability

All data generated or analyzed during the study are included in this published article.

## Supplementary Materials

The following supporting information can be downloaded at: https://www.mdpi.com/article/10.3390/jcm12103546/s1, Table S1: Classes of antibiotics used for treatment within 7 days after PE diagnosis.
