# Supplementary material for: Acute Infections and Inflammatory Biomarkers in Patients with Acute Pulmonary Embolism
Source: J Clin Med. 2023 May 18;12(10):3546. doi: 10.3390/jcm12103546 (PMC10219100; doi:10.3390/jcm12103546)
Supplement: Supplementary file 1 [file jcm-12-03546-s001.zip › jcm-2320466-supplementary.pdf]

Journal of
Clinical Medicine

MDPI

Supplementary materials

Acute Infections and Inflammatory Biomarkers in Patients with Acute Pulmonary Embolism

Ann-Sophie Eggers [1,2,*], Alaa Hafian [3], Markus H. Lerchbaumer [4], Gerd Hasenfuß [3,5], Karl Stangl [2,6], Burkert Pieske [7], Mareike Lankeit [3] and Matthias Ebner [2,6]

[1] Department of Cardiology, Angiology and Intensive Care Medicine, Charité Campus Virchow-Klinikum Mittelallee, German Heart Center of the Charité—University Medicine Berlin, 13353 Berlin, Germany
[2] German Center for Cardiovascular Research (DZHK), Partner Site Berlin, 10785 Berlin, Germany
[3] Clinic of Cardiology and Pneumology, University Medical Center Göttingen, 37075 Goettingen, Germany
[4] Department of Radiology, Campus Charité Mitte (CCM), Charité—University Medicine Berlin, 10117 Berlin, Germany
[5] German Center for Cardiovascular Research (DZHK), Partner Site Goettingen, 37075 Goettingen, Germany
[6] Department of Cardiology, Angiology and Intensive Care Medicine, Charité Campus Mitte, German Heart Center of the Charité—University Medicine Berlin, 10117 Berlin, Germany
[7] Independent Researcher, 13353 Berlin, Germany
* Correspondence: ann-sophie.eggers@dhzc-charite.de

Table S1 Classes of antibiotics used for treatment within 7 days after PE diagnosis

Any antibiotic treatment	347
Penicillins	244 (70.3%)
Cephalosporins	44 (12.7%)
Carbapenems	11 (3.2%)
Fluoroquinolones	63 (18.2%)
Macrolides	64 (18.4%)
Gylcopeptides	5 (1.4%)
Others	27 (7.8%)
